# Evidence for perinatal and child health care guidelines in crisis settings: can Cochrane help?

**DOI:** 10.1186/1471-2458-10-170

**Published:** 2010-03-29

**Authors:** Tari J Turner, Hayley Barnes, Jane Reid, Marie Garrubba

**Affiliations:** 1 Monash Institute of Health Services Research, Monash University, Locked Bag 29, Clayton 3168 Australia; 2 Centre for Clinical Effectiveness, Southern Health, Locked Bag 29, Clayton 3168 Australia; 3 previously of the Australasian Cochrane Centre, Monash University, Locked Bag 29, Clayton 3168 Australia

## Abstract

**Background:**

It is important that healthcare provided in crisis settings is based on the best available research evidence. We reviewed guidelines for child and perinatal health care in crisis situations to determine whether they were based on research evidence, whether Cochrane systematic reviews were available in the clinical areas addressed by these guidelines and whether summaries of these reviews were provided in Evidence Aid.

**Methods:**

Broad internet searches were undertaken to identify relevant guidelines. Guidelines were appraised using AGREE and the clinical areas that were relevant to perinatal or child health were extracted. We searched The Cochrane Database of Systematic Reviews to identify potentially relevant reviews. For each review we determined how many trials were included, and how many were conducted in resource-limited settings.

**Results:**

Six guidelines met selection criteria. None of the included guidelines were clearly based on research evidence. 198 Cochrane reviews were potentially relevant to the guidelines. These reviews predominantly addressed nutrient supplementation, breastfeeding, malaria, maternal hypertension, premature labour and prevention of HIV transmission. Most reviews included studies from developing settings. However for large portions of the guidelines, particularly health services delivery, there were no relevant reviews. Only 18 (9.1%) reviews have summaries in Evidence Aid.

**Conclusions:**

We did not identify any evidence-based guidelines for perinatal and child health care in disaster settings. We found many Cochrane reviews that could contribute to the evidence-base supporting future guidelines. However there are important issues to be addressed in terms of the relevance of the available reviews and increasing the number of reviews addressing health care delivery.

## Background

Populations in crisis settings such as those resulting from natural disasters like earthquake, famine and flood, as well as man-made disasters like civil war, are at substantial risk of trauma and illness. The risks of disease and death are particularly increased for infants, children and women giving birth. Health care providers in disaster settings work with limited infrastructure, equipment, supplies and training. Given the constrained resources with which they work, health care providers in these settings cannot afford to waste time or money on ineffective or harmful treatments.

Organisations working in disaster settings are developing an awareness of the need for their practice to be based on evidence of effectiveness. Clinical practice guidelines (CPGs) and other research-based tools are increasingly being used to support effective decision-making, and many humanitarian aid organisations have developed their own guidelines or adapted World Health Organization (WHO) guidelines. However, it has been suggested that there is a lack of evidence-based guidelines for child and perinatal health care in crisis situations [1], and WHO guidelines have been criticised for not being appropriately evidence-based [2].

The Cochrane Collaboration supports development and dissemination of high quality, systematic reviews of health research. After the devastating tsunami affecting the Pacific Rim in December 2004, The Cochrane Collaboration aimed to contribute to the relief effort by highlighting evidence relevant to people making decisions about health care in crisis situations through a website called Evidence Aid http://www.evidenceaid.org. Evidence Aid brings together information from Cochrane reviews on the effects of health care interventions relevant to the aftermath of a natural disaster or other large scale health care emergency. It aims to provide quick access to reliable information about which interventions work, which don't work, and which might be harmful.

In late 2007 The Cochrane Collaboration funded an evaluation of the effectiveness of Evidence Aid, which included an exploration of the ways in which Evidence Aid could contribute to meeting the health information needs of humanitarian organisations. In parallel with the evaluation of Evidence Aid, we reviewed existing guidelines for child and perinatal health care in crisis situations to determine what areas of clinical practice were addressed by these guidelines, and whether summaries of Cochrane systematic reviews relevant to these clinical areas were provided in Evidence Aid.

In this study we had several aims;

• to identify existing guidelines for perinatal and child health in crisis settings and examine whether these guidelines were explicitly based on research evidence,

• to determine whether relevant Cochrane systematic reviews were available in the clinical areas addressed by these guidelines and to ascertain whether summaries of these reviews were provided in Evidence Aid.

## Methods

### Identifying Existing Guidelines

#### Search Strategy

We undertook a broad search to identify existing guidelines for child and perinatal health care in crisis situations. We searched guideline websites, websites of humanitarian organisations and aid networks, and the internet using Google, with search terms including (emergency OR disaster OR crisis) AND (guideline OR evidence OR handbook OR manual).

Guideline websites included:

• US National Guidelines Clearinghouse http://www.guidelines.gov

• Guidelines International Network http://www.g-i-n.net,

• The Scottish Intercollegiate Guidelines Network http://www.sign.ac.uk

• The National Institute for Health and Clinical Excellence, http://www.nice.org.uk,

• The New Zealand Guidelines Group http://www.nzgg.org.nz

• The National Health and Medical Research Council http://www.nhmrc.gov.au

Humanitarian organisations and aid networks websites included:

• The World Health Organization http://www.who.int

• United Nations Office for the Coordination of Humanitarian Affairs http://ochaonline.un.org/

• The International Committee of the Red Cross http://www.icrc.org,

• Medecins Sans Frontieres http://www.msf.org,

• CARE http://www.care.org

• SPHERE http://www.sphereproject.org

• ReliefWeb http://www.reliefweb.int.

#### Selection Criteria

To be included guidelines had to be explicitly developed to provide guidance on health care interventions for child and perinatal conditions in crisis situations. Guidelines which provided policy, training or other guidance, but did not provide recommendations to guide decisions on provision of particular health care interventions were excluded as were guidelines that were not specifically developed to be implemented in crisis or emergency settings. We only included guidelines published in English. Selection criteria were applied independently by four reviewers, and disagreements resolved through discussion.

#### Quality Appraisal

As recommended by the AGREE Collaboration, included guidelines were appraised by four reviewers using the AGREE criteria, which are the standard for appraising the quality of evidence-based guidelines [3]. Guidelines were first assessed to determine whether they were based on systematic searches, and whether guideline recommendations were clearly linked to research evidence (AGREE criteria 8 & 12). Guidelines that did not meet these basic criteria of evidence-based guidelines were not further appraised. Guidelines that met these initial criteria were then fully appraised using the AGREE instrument.

#### Data Extraction

Two reviewers created a list of the clinical areas (disease/condition, symptom or intervention) addressed by each included guideline that were relevant to perinatal or child health.

### Identifying Relevant Cochrane Reviews

#### Search Strategy

For each clinical area extracted from the guidelines, we searched The Cochrane Database of Systematic Reviews http://www.thecochranelibrary.com Issue 2, 2008; using simple text word and MeSH searches of the title, abstract and keywords, to identify potentially relevant reviews. We searched only for Cochrane systematic reviews as this was part of a project exploring how Evidence Aid, which was developed by the Cochrane Collaboration and provides summaries of Cochrane systematic reviews, could contribute to meeting the health information needs of humanitarian organisations.

#### Data Extraction

For each potentially relevant review, one reviewer read the 'Description of Studies' section, 'Characteristics of Included Studies' table and 'References' section to determine how many trials were included in the review, and how many of these trials were conducted in a developing setting.

## Results

Fourteen potentially relevant guidelines were identified and six guidelines met selection criteria [4-9] (Tables 1 and 2). Of these six guidelines, three focused on feeding and nutrition for infants, children and/or pregnant and lactating women in emergencies [4,7,8], one addressed provision of antenatal care in crisis settings[5] and two covered all aspects of care for children in emergencies [6,9].

**Table 1:** Included guidelines

Title	Date	Developer
Manual for the health care of children in humanitarian emergencies [9]	2008	WHO

Infant and Young Child Feeding in Emergencies [4]	2007	IFE Core Group

Community-based Therapeutic Care (CTC) A Field Manual [7]	2006	Valid International

Antenatal Guidelines For Primary Health Care In Crisis Conditions [5]	2005	ICRC

Guiding principles for feeding infants and young children during emergencies [8]	2004	WHO

Emergency Field Handbook A Guide For UNICEF Staff [6]	Unclear	UNICEF

**Table 2:** Excluded guidelines

Title	Date	Developer	Reason for exclusion
Clinical Guidelines: Diagnosis and Treatment Manual for curative programs in hospitals and dispensaries [10]	2007	MSF	Not crisis setting

Obstetrics in Remote Settings: Practical guide for non-specialized health professionals [11]	2007	MSF	Not crisis setting

Pregnancy, Childbirth, Postpartum and Newborn Care: A guide for essential practice [12]	2006	WHO	Not crisis setting

Diarrhoea Treatment Guidelines Clinic-Based Healthcare Workers [13]	2005	MOST	Not crisis setting

Care of the Newborn Reference Manual [14]	2004	Save the Children Federation	Not crisis setting

Food and Nutrition Needs in Emergencies [15]	2002	UNHCR, UNICEF, WFP and WHO	Scope covered by more recent publication from the same organisations

The management of nutrition in major emergencies [16]	2000	WHO	Scope covered by more recent publication from the same organisations

Acute Respiratory Infections In Children [17]	Unclear	WHO	Not crisis setting

However, none of the included guidelines were explicitly based on systematic searches of research literature, or had recommendations that were clearly linked to research evidence (AGREE criteria 8 & 12). As a result none of the guidelines were assessed using the full AGREE instrument. One guideline did not include any references, one included references in a "Further reading" section and three included references, but these were not directly linked to the recommendations of the guideline. None of the guidelines included any Cochrane or other systematic reviews as references. None of the included guidelines described the methods by which they were developed.

Table 3 contains the reference numbers of the potentially relevant Cochrane systematic reviews and protocols for each guideline; the titles of these reviews and protocols are matched to the relevant guideline sections in Additional File 1 Table S1.

**Table 3:** References for Cochrane systematic reviews and protocols potentially relevant to each of the guidelines

Guideline	Cochrane systematic reviews	Protocols for reviews	Reviews with no included studies from developing settings
**Infant and Young Child Feeding in Emergencies **[4]	[18-31]	[32-37]	[21,24]

**Community-based Therapeutic Care (CTC) A Field Manual **[7]	[20,23,26,38-69]	[32,34,35,37,70-77]	[66] Unclear: [60-62]

**Guiding principles for feeding infants and young children during emergencies **[8]	[18,19,22,23,25-28,30,31,44,48,49,53,58,60-62,64-69,78-86]	[32,34,35,37,71,73,75,87-89]	[66,79], Unclear: [60-62,81].

**Antenatal Guidelines For Primary Health Care In Crisis Conditions **[5]	[25,27,28,30,31,48,60,61,64,90-194]	[195-206]	[91,92,95,99,101,107,108,116,117,121,125,127,128,130,134,135,138,141,147,151,162,163,167,171,172,176,177,179,180,182,184,186,192,194] Unclear: [60,61,90,96,98,100,106,109-111,114,119,129,131,132,140,146,149,150,157,158,166,173,175,188,193]

**Emergency Field Handbook: A Guide For UNICEF Staff **[6]	[18,19,22,26,27,39-43,45-48,50-54,56-59,61-63,65-69,78,83-85,105,191,207-231]	[35,36,73-75,77,87,88,232-244]	[66,207,213,222-224,230], Unclear: [61,62].

**Manual for the health care of children in humanitarian emergencies**[9]	[18,22,23,26,28,30,31,39-43,45-47,53,54,56,57,59,62,78,83-86,105,207,208,210,215,216,218,222,223,225,226,228-230,245-257]	[36,37,233,235,237,238,241,258]	[207,223,230,250] Unclear: [62,248,255]

The "Infant and Young Child Feeding in Emergencies" guideline [4] produced in 2007 by the Infant and Young Child Feeding in Emergencies (IFE) Core Group provides guidance on how to feed infants and children in crisis settings. There were 14 Cochrane systematic reviews and six protocols for reviews that were potentially relevant to this guideline, predominantly addressing nutrient supplementation, support for breastfeeding and prevention of mother to child HIV transmission. All except two of the reviews include some studies from developing settings. However for large portions of the guideline, particularly those focusing on procurement and distribution of infant formula, and coordinating, monitoring and assessing the success of feeding programs, there were no relevant reviews in The Cochrane Library.

For the Valid International Guideline "Community-based Therapeutic Care (CTC) A Field Manual" [7], which focuses on nutritional support for children, pregnant and lactating women, there were 35 potentially relevant Cochrane systematic reviews and 12 protocols. All of the reviews except one appeared to include studies from developing settings, however in three reviews the study settings were unclear. The potentially relevant reviews focused primarily on the effectiveness of supplementation with particular nutrients and choice of treatment for malaria. There were no relevant reviews addressing assessing malnutrition, planning, establishing, monitoring or evaluating a nutrition supplement program, which makes up the majority of the field manual.

There were 34 Cochrane reviews and 10 protocols potentially relevant to the World Health Organization's "Guiding principles for feeding infants and young children during emergencies" [8] guideline. As with the previous two guidelines, these reviews were primarily related to nutritional supplementation for infants and their mothers, as well as support for establishment and maintenance of breastfeeding. All but two trials appeared to include studies from developing settings, however in four reviews the study settings were unclear.

For the guideline addressing antenatal care "Antenatal Guidelines For Primary Health Care In Crisis Conditions" [5] produced by the International Committee of the Red Cross, there were 114 Cochrane systematic reviews and 12 protocols that were potentially relevant. The majority of these reviews addressed clinical management of hypertension, pre-eclampsia and eclampsia; or management of premature labour. For 34 of these reviews there were no included studies from developing settings, and in an additional 25 it was unclear how many studies were conducted in developing settings. The proportion of potentially relevant reviews where the included studies were all conducted in developed settings, or the setting was unclear was much larger for this guidelines than the others.

The "Emergency Field Handbook: A Guide For UNICEF Staff" [6] produced by UNICEF covers provision of protection and assistance to children and women in emergencies including: assessment, co-ordination, program delivery, security, communication, logistics and human resources. Sixty-two Cochrane systematic reviews and 21 protocols were potentially relevant to the content of this handbook; however, almost all of these were relevant to the section 'Health and Nutrition' which constitutes only approximately 20 percent of the content of the handbook. Very few Cochrane reviews were relevant to the rest of the content. There were no studies from developing settings in seven of the potentially relevant Cochrane reviews, and in another two reviews the setting of the included studies was unclear.

The WHO "Manual for the health care of children in humanitarian emergencies"[9] aims to provide guidance on the care of children in the acute and chronic phases of an emergency, where there are no inpatient hospital facilities. The content included diarrhoea and dehydration, cough or difficulty breathing, fever, malnutrition, anaemia, injuries, burns, poisoning, immunization and other public health measures, prevention of HIV Infection in children, and mental health and psychosocial support. There were 56 Cochrane reviews and eight protocols relevant to the content of this guideline, addressing much of the guideline content. However few reviews or protocols were found for topics on injury management, malnutrition, burns, or emergency assessment and triage. Research from developing settings was included in all but seven of the potentially relevant Cochrane reviews.

Of the total of 198 Cochrane systematic reviews that were identified as being potentially relevant to the content of the included guidelines, 18 (9.1%) have summaries in Evidence Aid. Links to the full text of the reviews are provided for a further twelve (6.1%).

## Discussion

Echoing what has been found previously [1], we were unable to identify any explicitly evidence-based guidelines to support decision-making around perinatal and child health care in crisis settings. The limited resources available and the enormous proportion of the burden of illness and death resulting from disasters borne by this portion of the population, mean that ensuring care delivery is evidence-based is vitally important for this setting.

It is possible to imagine a world where guidelines in all areas of clinical practice would provide recommendations based on systematic reviews of the best available evidence, integrated with clinical expertise and patient preferences. Cochrane reviews would be available to answer every clinical question, and after searching the Cochrane Database of Systematic Reviews, guidelines would be written, and we could be confident that the recommended care was based on a solid foundation of research evidence. However this ideal is unlikely to ever be achieved. Existing evidence-based guideline development methods are time-consuming and resource-intensive and often produce guidelines which are only applicable in a narrow range of contexts. Guideline developers do not always search for the best available research evidence, or do not document the methods used so that the evidence base on which the guidelines are developed cannot be established. Cochrane systematic reviews are not available to cover every possible clinical question, and many do not address issues of applicability and generalisability. In many areas primary research is not available, or is not directly relevant to the guideline being developed. These problems are exacerbated by the fact that it is very difficult to carry out primary research in crisis settings, and that most health research is carried out in well resourced settings, to address the health concerns of well resourced populations. As a result there is less primary research available relevant to crisis settings and potentially fewer relevant systematic reviews.

In spite of the barriers to the production of systematic reviews relevant to crisis settings, our results demonstrate that there are Cochrane reviews available which have the potential to support health care decision-making in perinatal and child health in crisis settings. A large number of potentially relevant reviews were identified. The great majority of the identified reviews include some studies that were undertaken in resource-poor settings, however very few, if any, were conducted in crisis settings. The contribution Cochrane reviews can make in the crisis setting is further limited in a number of important ways.

The Cochrane Database of Systematic Reviews includes few reviews on health system issues, focusing more on reviews of clinical interventions. While we only included guidelines that addressed at least one area of clinical practice, several of the included guidelines had substantial sections addressing establishing, prioritising or evaluating healthcare programs, and there is a clear need for evidence to support decision-making in these areas. The Cochrane Collaboration's contribution to synthesising research about health care delivery systems is currently limited, though may increase through the work of the Effective Practice and Organisation of Care group within the Collaboration. The critical need for this kind of evidence was also identified in our interviews with potential users of Evidence Aid as part of the broader Evidence Aid evaluation.

The large number of protocols for systematic reviews that are potentially relevant to the included guidelines suggests that the Cochrane Collaboration's contribution to the evidence-base of guidelines for perinatal and child health in crisis settings is likely to grow into the future. However the majority of these protocols address areas similar to those addressed in existing reviews, and the reviews arising from these protocols will not address the gap in evidence relating to health care delivery systems. This gap might be addressed by establishing processes to prioritise reviews in these areas of identified need and substantial morbidity and mortality.

Also, while The Cochrane Collaboration is developing methods for reviews of diagnostic studies, the Cochrane Database of Systematic Reviews does not currently include reviews of diagnosis, but only therapy. As a result, Cochrane reviews cannot inform decisions about diagnostic approaches, limiting their potential contribution to clinical practice guidelines.

Several issues impact on the potential relevance of the Cochrane reviews identified. One is the context in which most of the primary studies included in systematic reviews are conducted. The majority of these primary studies are undertaken in stable, developed countries, rather than unstable, resource-poor settings, where large scale emergencies are most common, and have the largest impact on increasing morbidity and mortality. This was particularly clear in the Cochrane reviews evaluating the impact of various forms of dietary supplementation. In many of these reviews the primary studies were not carried out in populations, like those seen in crisis situations, where dietary deficiencies are common. The effects of the supplements evaluated are likely to be very different in these settings, compared to those in which the studies were largely carried out. This highlights the importance of documenting the context of primary research studies included in systematic reviews.

When evidence-based guidelines make recommendations for practice, they often use evidence which was generated in contexts that are different from the one in which the guideline will be applied. In some situations evidence is available that was generated in a very similar context. However where this type of immediately relevant evidence is not available, guideline developers rely on other evidence, and draw conclusions about whether and/or how it is likely to apply in their context. The degree to which the context impacts on the applicability of the research varies with the nature of the clinical practice.

It is difficult to tightly define terms like 'crisis', 'disaster' and 'emergency'. We chose a very broad definition "crisis settings such as those resulting from natural disasters like earthquake, famine and flood, as well as man-made disasters like civil war", designed to be inclusive, but to differentiate from what might be considered 'stable' situations in resource-poor settings. However the threshold between crisis and non-crisis is essentially arbitrary and there will always be grey areas. There are many examples where 'normal' conditions in resource-poor settings have deteriorated so that they could reasonably be described as crisis situations and also where crisis situations have become so chronic and entrenched that they are essentially 'stable'.

In developing guidelines for crisis situations, there will be times when it is appropriate to base recommendations on evidence established in stable situations and other times when the evidence must be generated in crisis settings to be relevant to the practice in question. Similarly some guidelines developed for use in stable situations may be relevant and appropriate for use in crisis situations. The nature of crisis situations varies widely. Crises arise from many different causes, and take place in contexts with widely varying health infrastructure, staff availability, geography and underlying health problems, etc. All of these factors will affect the relevance of research and guidelines generated in other settings.

A second factor impacting on the potential relevance is the availability of the interventions evaluated in the Cochrane reviews. While we attempted to include only those reviews addressing interventions which were potentially feasible in crisis settings (guided by the interventions recommended by the guidelines themselves); in the aftermath of emergencies, clinicians are often working with a very limited toolkit of equipment and drugs, and these may vary from one situation to another. As a result, some of the reviews which we believe might be potentially relevant may recommend interventions which are not affordable or practical in some crisis settings. However, the large number of potentially relevant Cochrane reviews identified suggests that, even taking these factors into account, Cochrane reviews can still make a substantial contribution to supporting health care decision-making in these contexts.

A relatively small percentage of the Cochrane reviews identified as being potentially relevant to informing guidelines for perinatal and child health in crisis settings currently have summaries in Evidence Aid. So the results of these reviews are only available to those who have access to the full text of The Cochrane Library. As a result The Cochrane Collaboration is missing an opportunity to provide evidence to support humanitarian organisations developing guidelines in these areas. This gap may result from a lack of awareness that the results of these reviews would potentially be valuable in these settings, or, perhaps more likely, from time and resource limitations on the production of the Evidence Aid summaries, a process that requires a substantial investment of time and intellect.

This review is limited by our focus on guidelines in the field of perinatal and child health, which may not be representative of the state of guidelines in other areas of emergency health care. However infants, children and pregnant women are particularly at risk in the aftermath of emergencies, and so we feel this focus is reasonable, if largely pragmatic. The review was also limited to those guidelines that were available in English on the internet. High quality evidence-based guidelines relevant to this area may have been developed but not made publicly available. We chose to focus on the health issues surrounding crisis situations, however we acknowledge that these situations also involve environmental, engineering and social problems. Our focus was on health issues as this our area of expertise and this is also the area in which the methods for developing clinical practice guidelines have been established. The study is also limited to assessing the reviews that are available at the current time, which may be different from those available at the time the guidelines were developed, which was not always clear from the guidelines. However the included guidelines were all developed relatively recently, and this study highlights that there is evidence relevant to this area of clinical practice that could be used as the basis of future guideline development.

## Conclusion

We were unable to identify any explicitly evidence-based guidelines to support decision-making around perinatal and child health care in disaster settings. We found that there are many Cochrane reviews available that have the potential to contribute to the evidence-base supporting guidelines for perinatal and child health in disaster settings in the future, and that most of these reviews include some studies conducted in developing settings. However there are also important issues to be addressed in terms of the relevance of the available reviews and increasing the number of reviews addressing questions of health care delivery.

## Competing interests

TT undertook a PhD supervised by the Director of the Australasian Cochrane Centre (ACC) which included an evaluation of Evidence Aid, funded through a grant from The Cochrane Collaboration Steering Group Discretionary Fund. HB was previously employed by the ACC. JR and MG both work on projects, unrelated to this project, in which ACC is a collaborator.

## Authors' contributions

TT conceived of this project and developed the methodology and data extraction approach, which HB, JR and MG refined. TT and MG undertook the searches. TT, HB, JR and MG applied the AGREE criteria. TT undertook the data extraction with assistance from HB and JR. TT prepared the first draft of this article which HB, JR, MG and TT then revised. TT had full access to all the data in the study and had final responsibility for the decision to submit for publication. All authors read and approved the final manuscript

## Pre-publication history

The pre-publication history for this paper can be accessed here:

http://www.biomedcentral.com/1471-2458/10/170/prepub

## Supplementary Material

Additional file 1 **Table S1**. Details of potentially relevant Cochrane systematic reviews and protocols for each guideline Click here for file
