# Supplementary material for: Evidence for perinatal and child health care guidelines in crisis settings: can Cochrane help?
Source: BMC Public Health. 2010 Mar 29;10:170. doi: 10.1186/1471-2458-10-170 (PMC3091544; doi:10.1186/1471-2458-10-170)
Supplement: Additional file 1 — Table S1. Details of potentially relevant Cochrane systematic reviews and protocols for each guideline [file 1471-2458-10-170-S1.DOC]

# Additional Files

**Table S1. Details of potentially relevant Cochrane systematic reviews and protocols** for each guideline

| **Guideline Name** | Infant and Young Child Feeding in Emergencies |  |  |
| --- | --- | --- | --- |
| **Date** | 2007 |  |  |
| **Developer** | IFE Core Group |  |  |
| **Guideline section** | **Potentially Relevant Cochrane Reviews** | **No. of trials in review** | **No. from developing setting** |
| 1 Endorse or Develop Policies | nil |  |  |
| 2 Train Staff | nil |  |  |
| 3 Co-ordinate Operations | nil |  |  |
| 4 Assess and Monitor | nil |  |  |
| **5 Protect, Promote and Support Optimal Infant and Young Child Feeding with Integrated Multi-Sectoral Interventions** | |  |  |
| *5.1 Basic interventions* |  |  |  |
| General rations | Community-based supplementary feeding for promoting the growth of young children in developing countries [1] | 4 | 4 |
| Complementary feeding | Iron therapy for improving psychomotor development and cognitive function in children under the age of three with iron deficiency anaemia [2] | 7 | 4 |
|  | Iron supplementation for iron deficiency anemia in children [3] | Protocol |  |
|  | Routine iron supplementation for preventing or treating iron-deficiency anaemia in children in malaria-endemic areas [4] | Protocol |  |
|  | Iron supplementation for improving psychomotor development and cognitive function in infants under the age of one [5] | Protocol |  |
|  | Iron supplementation for reducing morbidity and mortality in children with HIV [6] | Protocol |  |
| Micronutrients | Multiple-micronutrient supplementation for women during pregnancy [7] | 9 | 8 |
|  | Supplementation with two or more micronutrients versus single micronutrient supplementation or placebo in young children [8] | Protocol |  |
| Newborn registration | nil |  |  |
| Caregiver support | nil |  |  |
| *5.2 Technical interventions* |  |  |  |
| Training | nil |  |  |
| Service development and integration | nil |  |  |
| HIV and AIDS considerations | Formula milk versus donor breast milk for feeding preterm or low birth weight infants [9] | 8 | 0 |
|  | Formula milk versus maternal breast milk for feeding preterm or low birth weight infants [10] | 0 | 0 |
|  | Interventions to decrease the risk of mother-to-child transmission of HIV-1 through breast milk [11] | Protocol |  |
|  | Support for breastfeeding mothers [12] | 34 | 9 |
|  | Optimal duration of exclusive breastfeeding [13] | 22 | 11 |
|  | Traditional birth attendant training for improving health behaviours and pregnancy outcomes [14] | 4 | 4 |
|  | Condom effectiveness in reducing heterosexual HIV transmission [15] | 14 | 4 |
|  | Antiretrovirals for reducing the risk of mother-to-child transmission of HIV infection [16] | 14 | 14 |
|  | Efficacy and safety of cesarean delivery for prevention of mother-to-child transmission of HIV-1 [17] | 6 | 1 |
|  | Vitamin A supplementation for reducing the risk of mother-to-child transmission of HIV infection [18] | 4 | 4 |
|  | Vaginal disinfection for preventing mother-to-child transmission of HIV infection [19] | 2 | 2 |
| **6 Minimise the Risks of Artificial Feeding** | |  |  |
| 6.1 Handling BMS donations and supplies | nil |  |  |
| 6.2 Establish and implement criteria for targeting and use | nil |  |  |
| *6.3 Control of procurement donor agency responsibilities* | |  |  |
| Type and source of formula | nil |  |  |
| Labelling | nil |  |  |
| Supply conditions | nil |  |  |
| Bottles and teats | Cup feeding versus other forms of supplemental enteral feeding for newborn infants unable to fully breastfeed [20] | 4 | 1 |
| 6.4 Control of management and distribution | nil |  |  |

| **Guideline Name** | Community-based Therapeutic Care (CTC) A Field Manual |  |  |
| --- | --- | --- | --- |
| **Date** | 2006 |  |  |
| **Developer** | Valid International |  |  |
| **Guideline section** | **Potentially Relevant Cochrane Reviews** | **No. of trials in review** | **No. from developing setting** |
| 1. Introduction | nil |  |  |
| 2. The CTC model | nil |  |  |
| 3. CTC in context | nil |  |  |
| 4. Planning and design | nil |  |  |
| 5. Community mobilisation | nil |  |  |
| **6. Supplementary feeding programme** | nil |  |  |
| 6.1 Planning | nil |  |  |
| 6.2 Target Group and Admission/Discharge Criteria | nil |  |  |
| *6.3 Treatment Protocols and Procedures* |  |  |  |
| 6.3.1 Nutritional Management of Moderately Malnourished Children | Community-based supplementary feeding for promoting the growth of young children in developing countries [1] | 4 | 4 |
| 6.3.2 Medical Management of Moderately Malnourished Children | Deworming drugs for treating soil-transmitted intestinal worms in children: effects on growth and school performance [21] | 34 | 34 |
|  | Vitamin A for preventing acute lower respiratory tract infections in children up to seven years of age [22] | 9 | 7 |
|  | Neonatal vitamin A supplementation for the prevention of mortality and morbidity in term neonates in  developing countries [23] | Protocol |  |
|  | Vaccines for measles, mumps and rubella in children [24] | 31 | Not described |
|  | Iron therapy for improving psychomotor development and cognitive function in children under the age of three with iron deficiency anaemia [2] | 7 | 4 |
|  | Iron supplementation for iron deficiency anemia in children [3] | Protocol |  |
|  | Routine iron supplementation for preventing or treating iron-deficiency anaemia in children in malaria-endemic areas [4] | Protocol |  |
|  | Iron supplementation for improving psychomotor development and cognitive function in infants under the age of one [5] | Protocol |  |
|  | Iron supplementation for reducing morbidity and mortality in children with HIV [6] | Protocol |  |
|  | Iodised salt for preventing iodine deficiency disorders [25] | 6 | 4 |
|  | Iodine supplementation for preventing iodine deficiency disorders in children [26] | 26 | 22 |
|  | Vitamin D supplementation for improving bone mineral density in children [27] | Protocol |  |
|  | Calcium supplementation for improving bone mineral density in children [28] | 19 | 4 |
|  | Zinc supplementation for the prevention of pneumonia in children aged 2 months to 59 months [29] | Protocol |  |
| 6.3.3 Monitoring and Transfer of Moderately Malnourished Children | Growth monitoring in children [30] | 2 | 2 |
| 6.3.4 Nutritional and Medical Management of Moderately Malnourished Pregnant and Lactating Women | Zinc supplementation for improving pregnancy and infant outcome [31] | 17 | 13 |
|  | Energy and protein intake in pregnancy [32] | 23 | At least 9. Not well described |
|  | Multiple-micronutrient supplementation for women during pregnancy [7] | 9 | 8 |
|  | Vitamin A supplementation during pregnancy [33] | 5 | 3 |
|  | Vitamin C supplementation in pregnancy [34] | 5 | 3 |
|  | Vitamin D supplementation in pregnancy [35] | 2 | 0 |
|  | Vitamin E supplementation in pregnancy [36] | 4 | 2 |
|  | Treatments for iron-deficiency anaemia in pregnancy [37] | 17 | Not described |
|  | Vitamin A supplementation for breastfeeding mothers [38] | Protocol |  |
|  | Effects of interventions for helminthic infections in pregnancy [39] | Protocol |  |
|  | Effects of routine oral iron supplementation with or without folic acid for women during pregnancy [40] | 40 | At least 15. Not well described |
|  | Treatment for women with postpartum iron deficiency anaemia [41] | 6 | Not described |
|  | Folate supplementation in pregnancy [42] | Protocol |  |
|  | Calcium supplementation (other than for preventing or treating hypertension) for improving pregnancy and infant outcomes [43] | Protocol |  |
| 6.3.5 Demonstration of Food Preparation | nil |  |  |
| 6.4 Data Collection and Monitoring | nil |  |  |
| **7. Outpatient therapeutic programme** |  |  |  |
| 7.1 Planning | nil |  |  |
| 7.2 Target Group and Admission/Discharge Criteria | nil |  |  |
| *7.3 Treatment Protocols and Procedures* |  |  |  |
| 7.3.1 Nutritional Treatment | As above |  |  |
| 7.3.2 Medicines | As above |  |  |
| 7.3.3 Health Education | Hand washing for preventing diarrhoea [44] | 14 | 6 |
|  | Face washing promotion for preventing active trachoma [45] | 2 | 2 (1 in north Aust) |
|  | Environmental sanitary interventions for preventing active trachoma [46] | 4 | 4 |
| 7.3.4 Follow-Up | nil |  |  |
| 7.3.5 Links to Other Programmes | nil |  |  |
| 7.4 Data Collection and Monitoring | nil |  |  |
| **8. Stabilisation Care** |  |  |  |
| 8.1 Planning | nil |  |  |
| 8.2 Target Group and Admission/Discharge Criteria | nil |  |  |
| *8.3 Treatment Protocols and Procedures* |  |  |  |
| 8.3.1 On Admission | As above |  |  |
| 8.3.2 Nutritional Treatment | As above |  |  |
| 8.3.3 Medical Treatment | As above |  |  |
| 8.3.4 Observation and Monitoring | nil |  |  |
| 8.3.5 Hygiene | nil |  |  |
| 8.3.6 On Discharge | nil |  |  |
| 8.4 Data Collection and Monitoring | nil |  |  |
| **9. Monitoring** | nil |  |  |
| **10. Evaluation** | nil |  |  |
| **11. Local production of RUTF** | nil |  |  |
| **12. Future developments** | nil |  |  |
| **Appendices** | Intramuscular arteether for treating severe malaria [47] | 2 | 2 |
|  | Sulfadoxine-pyrimethamine plus artesunate versus sulfadoxine-pyrimethamine plus amodiaquine for treating uncomplicated malaria [48] | 4 | 4 |
|  | Chlorproguanil-dapsone for treating uncomplicated malaria [49] | 6 | 6 |
|  | Artesunate plus mefloquine versus mefloquine for treating uncomplicated malaria [50] | 8 | 8 |
|  | Intra-rectal quinine for treating Plasmodium falciparum malaria [51] | 8 | 8 |
|  | Artesunate versus quinine for treating severe malaria [52] | 6 | 6 |
|  | High first dose quinine regimen for treating severe malaria [53] | 4 | 4 |
|  | Chloroquine or amodiaquine combined with sulfadoxine-pyrimethamine for treating uncomplicated malaria [54] | 4 | 4 |
|  | Artemether-lumefantrine (six-dose regimen) for treating uncomplicated falciparum malaria [55] | 9 | 9 |
|  | Artemether-lumefantrine (four-dose regimen) for treating uncomplicated falciparum malaria [56] | 7 | 7 |
|  | Atovaquone-proguanil for treating uncomplicated malaria [57] | 10 | 9 |
|  | Antibiotics for trachoma [58] | 15 | At least 7 |
|  | Antifungal agents for the treatment of mucocutaneous candidiasis in neonates and children [59] | Protocol |  |
|  | Interventions for treating scabies [60] | 20 | 17 |

| **Guideline Name** | Guiding principles for feeding infants and young children during emergencies |  |  |
| --- | --- | --- | --- |
| **Date** | 2004 |  |  |
| **Developer** | WHO |  |  |
| **Guideline section** | **Potentially Relevant Cochrane Reviews** | **No. of trials in review** | **No. from developing setting** |
| **Introduction** |  |  |  |
| **Breastfeeding** | Optimal duration of exclusive breastfeeding [13] | 22 | 11 |
| *Factors affecting successful breastfeeding* | Support for breastfeeding mothers [12] | 34 | 9 |
|  | Interventions in the workplace to support breastfeeding for women in employment [61] | 0 | 0 |
|  | Interventions for promoting the initiation of breastfeeding [62] | 7 | 2 |
|  | Community-based supplementary feeding for promoting the growth of young children in developing countries [1] | 4 | 4 |
|  | Lactational amenorrhea for family planning [63] | 12 | At least 7. Not well described |
|  | Antiretrovirals for reducing the risk of mother-to-child transmission of HIV infection [16] | 14 | 14 |
|  | Efficacy and safety of cesarean delivery for prevention of mother-to-child transmission of HIV-1 [17] | 6 | 1 |
|  | Vitamin A supplementation for reducing the risk of mother-to-child transmission of HIV infection [18] | 4 | 4 |
|  | Vaginal disinfection for preventing mother-to-child transmission of HIV infection [19] | 2 | 2 |
|  | Traditional birth attendant training for improving health behaviours and pregnancy outcomes [14] | 4 | 4 |
|  | Cup feeding versus other forms of supplemental enteral feeding for newborn infants unable to fully breastfeed [20] | 4 | 1 |
|  | Separate care for new mother and infant versus rooming-in for increasing the duration of breastfeeding [64] | Protocol |  |
|  | Treatments for suppression of lactation [65] | Protocol |  |
|  | Parent-training programmes for improving maternal psychosocial health [66] | 26 | Not described |
| *Other breast-milk feeding options* | Nil |  |  |
| Breastmilk substitutes | Cup feeding versus other forms of supplemental enteral feeding for newborn infants unable to fully breastfeed [20] | 4 | 4 |
| **Complementary feeding** | Early additional food and fluids for healthy breastfed full-term infants [67] | Protocol |  |
| Foods for older infants and young children | nil |  |  |
| Special problems | nil |  |  |
| Adjusting to change | nil |  |  |
| Inexperienced caregivers | nil |  |  |
| Factors related to children | nil |  |  |
| Feeding frequency | nil |  |  |
| Child/caregiver interaction | nil |  |  |
| General food aid | Community-based supplementary feeding for promoting the growth of young children in developing countries [1] | 4 | 4 |
| Using general food aid commodities to feed older infants and young children | nil |  |  |
| Possible inadequacies in general food aid commodities | Effects of routine oral iron supplementation with or without folic acid for women during pregnancy [40] | 40 | At least 15. Not well described |
|  | Treatment for women with postpartum iron deficiency anaemia [41] | 6 | Not described |
|  | Iron supplementation for iron deficiency anemia in children [3] | Protocol |  |
|  | Routine iron supplementation for preventing or treating iron-deficiency anaemia in children in malaria-endemic areas [4] | Protocol |  |
|  | Iron supplementation for improving psychomotor development and cognitive function in infants under the age of one [5] | Protocol |  |
|  | Iron supplementation for reducing morbidity and mortality in children with HIV [6] | Protocol |  |
|  | Iron therapy for improving psychomotor development and cognitive function in children under the age of three with iron deficiency anaemia [2] | 7 | 4 |
|  | Iodine supplementation for preventing iodine deficiency disorders in children [26] | 26 | 22 |
|  | Iodised salt for preventing iodine deficiency disorders [25] | 6 | 4 |
|  | Calcium supplementation for improving bone mineral density in children [28] | 19 | 4 |
|  | Interventions for the prevention of nutritional rickets in term born children [68] | 4 | 2 |
|  | Calcium supplementation (other than for preventing or treating hypertension) for improving pregnancy and infant outcomes [43] | Protocol |  |
| Blended foods | nil |  |  |
| Distribution of special commodities | nil |  |  |
| Supplementary food distribution | nil |  |  |
| Food from other sources | nil |  |  |
| Household food production | nil |  |  |
| Purchase and barter | nil |  |  |
| Natural food collection | nil |  |  |
| Safe food, safe feeding | Hand washing for preventing diarrhoea [44] | 14 | 6 |
| **Caring for caregivers** |  |  |  |
| All caregivers | Interventions to improve water quality for preventing diarrhoea [69] | 30 | 29 |
| Households with only one adult | nil |  |  |
| *Meeting women’s special needs* | Zinc supplementation for improving pregnancy and infant outcome [31] | 17 | 13 |
|  | Energy and protein intake in pregnancy [32] | 23 | At least 9. Not well described |
|  | Multiple-micronutrient supplementation for women during pregnancy [7] | 9 | 8 |
|  | Vitamin A supplementation during pregnancy [33] | 5 | 3 |
|  | Vitamin C supplementation in pregnancy [34] | 5 | 3 |
|  | Vitamin D supplementation in pregnancy [35] | 2 | 0 |
|  | Vitamin E supplementation in pregnancy [36] | 4 | 2 |
|  | Treatments for iron-deficiency anaemia in pregnancy [37] | 6 | Not described |
|  | Vitamin A supplementation for breastfeeding mothers [38] | Protocol |  |
|  | Effects of routine oral iron supplementation with or without folic acid for women during pregnancy [40] | 40 | At least 15. Not well described |
|  | Folate supplementation in pregnancy [42] | Protocol |  |
|  | Calcium supplementation (other than for preventing or treating hypertension) for improving pregnancy and infant outcomes [43] | Protocol |  |
| Rape prevention and response | nil |  |  |
| Other trauma | nil |  |  |
| **Protecting children** |  |  |  |
| The prenatal and postpartum periods | Vaccines for measles, mumps and rubella in children [24] | 31 | Not described |
|  | Vitamin A supplementation for breastfeeding mothers [38] | Protocol |  |
|  | Treatment for women with postpartum iron deficiency anaemia [41] | 6 | Not described |
| Illness | nil |  |  |
| The physical environment | Early skin-to-skin contact for mothers and their healthy newborn infants [70] | 30 | 7 |
|  | Interventions to prevent hypothermia at birth in preterm and/or low birthweight infants [71] | 6 | 1 |
|  | Kangaroo mother care to reduce morbidity and mortality in low birthweight infants [72] | 3 | 3 |
| **Malnutrition** |  |  |  |
| **The acute phase of emergencies** | nil |  |  |
| Suggestions for early interventions | nil |  |  |
| **Assessment, intervention and monitoring** | nil |  |  |
| Initial assessment and preparation for action | nil |  |  |
| Practical considerations | nil |  |  |
| Information | nil |  |  |
| Resources | nil |  |  |
| Communication | nil |  |  |
| Support networks | nil |  |  |
| Special programmes | nil |  |  |
| Community-based action | nil |  |  |
| Monitoring | nil |  |  |
| **Conclusion** |  |  |  |

| **Guideline Name** | Antenatal Guidelines For Primary Health Care In Crisis Conditions |  |  |
| --- | --- | --- | --- |
| **Date** | 2005 |  |  |
| **Developer** | ICRC |  |  |
| **Guideline section** | **Potentially Relevant Cochrane Reviews** | **No. of trials in review** | **No. from developing setting** |
| **Part I: Antenatal care programmes** | |  |  |
| Aims | nil |  |  |
| Setting up an antenatal care programme | nil |  |  |
| Monitoring an antenatal care programme | nil |  |  |
| IDRC Assistance | nil |  |  |
| Basic emergency equipment | nil |  |  |
| Health Education | Antenatal breastfeeding education for increasing breastfeeding duration [73] | Protocol |  |
|  | Education for contraceptive use by women after childbirth [74] | 3 | 2 |
|  | Information for pregnant women about caesarean birth [75] | 2 | 0 |
|  | Interventions for promoting smoking cessation during pregnancy [76] | 64 | 1 |
|  | Prenatal education for congenital toxoplasmosis [77] | Protocol |  |
|  | Traditional birth attendant training for improving health behaviours and pregnancy outcomes [14] | 4 | 4 |
| **Part II : Antenatal consultations** | |  |  |
| Medical records | nil |  |  |
| Diagnosis of pregnancy and Calculation of term | nil |  |  |
| Physiological changes in pregnancy | nil |  |  |
| Initial medical assessment | nil |  |  |
| Standard antenatal consultation | nil |  |  |
| Cases to be referred for delivery | nil |  |  |
| **Emergency obstetric care** |  |  |  |
| General information | nil |  |  |
| Emotional and psychological support | Continuous support for women during childbirth [78] | 16 | 4 |
|  | Psychosocial and psychological interventions for treating antenatal depression [79] | 1 | 0 |
|  | Antidepressant prevention of postnatal depression [80] | 2 | 0 |
| Convulsions or loss of consciousness  *See below for pre-eclampsia and eclampsia* | Diazepam for treating tetanus [81] | 2 | 2 |
|  | Common antiepileptic drugs in pregnancy in women with epilepsy [82] | 7 | 0 |
|  | Drugs for treating uncomplicated malaria in pregnant women [83] | 6 | 6 |
| Fever | Antipyretic measures for treating fever in malaria [84] | 3 | 2 |
|  | Antibiotic regimens for endometritis after delivery [85] | 39 | 3 |
|  | Umbilical vein injection for management of retained placenta [86] | 12 | Not described |
|  | Antibiotic regimens for management of intraamniotic infection [87] | 2 | 0 |
| Respiratory distress | Nil |  |  |
| Vaginal bleeding | Interventions for tubal ectopic pregnancy [88] | 35 | 5 |
|  | Prophylactic antibiotics for manual removal of retained placenta in vaginal birth [89] | 0 | 0 |
|  | Treatment for primary postpartum haemorrhage [90] | 3 | 3 |
|  | Treatments for secondary postpartum haemorrhage [91] | 0 | 0 |
|  | Expectant care versus surgical treatment for miscarriage [92] | 5 | Not described |
|  | Interventions for suspected placenta praevia [93] | 3 | 1 |
|  | Interventions for treating placental abruption [94] | 0 | 0 |
|  | Antibiotics for incomplete abortion [95] | 1 | 1 |
|  | Medical treatment for early fetal death (less than 24 weeks) [96] | 24 | At least 5. Not well described |
|  | Progestogen for treating threatened miscarriage [97] | 2 | Not described |
|  | Uterine muscle relaxant drugs for threatened miscarriage [98] | 1 | 1 |
| Abdominal pain | As above |  |  |
| Prolapsed cord | Nil |  |  |
| Unsatisfactory progress in labour | Oxytocin versus placebo or no treatment for slow progress in the first stage of spontaneous labour [99] | Protocol |  |
| Trauma |  |  |  |
| Controlled Cord Traction | Fundal pressure versus controlled cord traction as part of the active management of the third stage of labour [100] | 0 | 0 |
|  | Active versus expectant management in the third stage of labour [101] | 5 | 0 |
| Uterine massage | Uterine massage for preventing postpartum haemorrhage [102] | 1 | 1 |
| Anaemia | Treatments for iron-deficiency anaemia in pregnancy [37] | 17 | Not described |
|  | Treatment for women with postpartum iron deficiency anaemia [41] | 6 | Not described |
|  | Effects of routine oral iron supplementation with or without folic acid for women during pregnancy [40] | 40 | At least 15. Not well described |
| Female genital mutilation | Nil |  |  |
| Fever | As above |  |  |
| Malaria | Drugs for preventing malaria in pregnant women [103] | 16 | 16 |
|  | Insecticide-treated nets for preventing malaria in pregnancy [104] | 5 | 5 |
|  | Drugs for treating uncomplicated malaria in pregnant women [83] | 6 | 6 |
|  | Intermittent preventive treatment regimens for malaria in HIV-positive pregnant women [105] | Protocol |  |
| Urinary tract infection | Treatments for symptomatic urinary tract infections during pregnancy [106] | 9 | 3 |
|  | Duration of treatment for asymptomatic bacteriuria during pregnancy [107] | 10 | 0 |
| *Hypertensive disorders in pregnancy* | Antioxidants for preventing pre-eclampsia [108] | 10 | At least 4. |
|  | Calcium supplementation during pregnancy for preventing hypertensive disorders and related problems [109] | 12 | 7 |
|  | Diuretics for preventing pre-eclampsia [110] | 5 | 0 |
|  | Exercise or other physical activity for preventing pre-eclampsia and its complications [111] | 2 | 0 |
|  | Altered dietary salt for preventing pre-eclampsia, and its complications [112] | 2 | 0 |
|  | Progesterone for preventing pre-eclampsia and its complications [113] | 2 | 0 |
|  | Rest during pregnancy for preventing pre-eclampsia and its complications in women with normal blood pressure [114] | 2 | 1 |
|  | Antiplatelet agents for preventing pre-eclampsia and its complications [115] | 59 | At least 16. |
|  | Chinese herbal medicine for the treatment of pre-eclampsia [116] | 0 | 0 |
|  | Garlic for preventing pre-eclampsia and its complications [117] | 1 | 0 |
|  | Nitric oxide for preventing pre-eclampsia and its complications [118] | 6 | 0 |
| Management of mild or severe chronic hypertension | Reduced salt intake compared to normal dietary salt, or high intake, in pregnancy [119] | 2 | 0 |
|  | Antihypertensive drug therapy for mild to moderate hypertension during pregnancy [120] | 46 | 12 |
| Management of pregnancy - induced hypertension | Drugs for treatment of very high blood pressure during pregnancy [121] | 24 | At least 13. |
| Mild pre-eclampsia | Magnesium sulphate and other anticonvulsants for women with pre-eclampsia [122] | 13 | At least 5. |
| Management of severe pre-eclampsia and eclampsia | Interventionist versus expectant care for severe pre-eclampsia before term [123] | 2 | 1 |
|  | Abdominal decompression for suspected fetal compromise/pre-eclampsia [124] | 3 | Not described |
|  | Low-dose dopamine for women with severe pre-eclampsia [125] | 1 | Not described |
|  | Corticosteroids for HELLP syndrome in pregnancy [126] | 5 | Not described |
|  | Magnesium sulphate versus diazepam for eclampsia [127] | 7 | 7 |
|  | Magnesium sulphate versus phenytoin for eclampsia [128] | 6 | 4 |
|  | Plasma volume expansion for treatment of pre-eclampsia [129] | 3 | 1 |
|  | Magnesium sulphate versus lytic cocktail for eclampsia [130] | 2 | 2 |
| *Loss or diminution of foetal movements* | Fetal movement counting for assessment of fetal wellbeing [131] | 4 | At least 1 |
| Foetal or neonatal death | Medical treatment for early fetal death (less than 24 weeks) [96] | 24 | At least 5. Not well described |
|  | Misoprostol for induction of labour to terminate pregnancy in the second or third trimester for women with a fetal anomaly or after intrauterine fetal death [132] | Protocol |  |
|  | Support for mothers, fathers and families after perinatal death [133] | 0 | 0 |
|  | Epinephrine for the resuscitation of apparently stillborn or extremely bradycardic newborn infants [134] | 0 | 0 |
| Foetal growth retardation | Bed rest in hospital for suspected impaired fetal growth [135] | 1 | Not described |
|  | Betamimetics for suspected impaired fetal growth [136] | 2 | Not described |
|  | Calcium channel blockers for potential impaired fetal growth [137] | 1 | Not described |
|  | Hormones for suspected impaired fetal growth [138] | 0 | 0 |
|  | Maternal nutrient supplementation for suspected impaired fetal growth [139] | 4 | Not described |
|  | Maternal oxygen administration for suspected impaired fetal growth [140] | 3 | 2 |
|  | Plasma volume expansion for suspected impaired fetal growth [141] | 0 | 0 |
| *Malpresentations* |  |  |  |
| Breech | Cephalic version by moxibustion for breech presentation [142] | 3 | 2 |
|  | Expedited versus conservative approaches for vaginal delivery in breech presentation [143] | 0 | 0 |
|  | External cephalic version for breech presentation before term [144] | 3 | Unclear |
|  | Planned caesarean section for term breech delivery [145] | 3 | 1 |
|  | Cephalic version by postural management for breech presentation [146] | 7 | Not described |
|  | External cephalic version for breech presentation at term [147] | 5 | Not described |
|  | Interventions to help external cephalic version for breech presentation at term [148] | 16 | Not described |
| Transverse | Hands and knees posture in late pregnancy or labour for fetal malposition (lateral or posterior) [149] | 3 | 1 |
| Pregnancy after rape / Rape of pregnant woman | Nil |  |  |
| Pregnancy in detention | Nil |  |  |
| Pregnancy in teenagers | Interventions for preventing unintended pregnancies among adolescents [150] | Protocol |  |
| Prelabour rupture of membranes | Antibiotics for prelabour rupture of membranes at or near term [151] | 2 | Not described |
|  | Planned early birth versus expectant management (waiting) for prelabour rupture of membranes at term (37 weeks or more) [152] | 12 | Not described |
|  | Prophylactic antibiotic administration in pregnancy to prevent infectious morbidity and mortality [153] | 6 | 3 |
|  | Planned early birth versus expectant management for women with preterm prelabour rupture of membranes prior to 37 weeks' gestation for improving pregnancy outcome [154] | Protocol |  |
| *Sexually transmitted diseases* |  |  |  |
| Viral hepatitis | Caesarean section versus vaginal delivery for preventing mother to infant hepatitis C virus transmission [155] | 0 | 0 |
|  | Hepatitis B immunisation for newborn infants of hepatitis B surface antigen-positive mothers [156] | 29 | 24 |
| HIV/AIDS | Antiretrovirals for reducing the risk of mother-to-child transmission of HIV infection [16] | 14 | 14 |
|  | Efficacy and safety of cesarean delivery for prevention of mother-to-child transmission of HIV-1 [17] | 6 | 1 |
|  | Vitamin A supplementation for reducing the risk of mother-to-child transmission of HIV infection[18] | 4 | 4 |
|  | Vaginal disinfection for preventing mother-to-child transmission of HIV infection [19] | 2 | 2 |
|  | Intermittent preventive treatment regimens for malaria in HIV-positive pregnant women [105] | Protocol |  |
| Gonorrhoea | Antibiotics for gonorrhoea in pregnancy [157] | 2 | Not described |
| Chlamydia trachomatis | Interventions for treating genital chlamydia trachomatis infection in pregnancy [158] | 11 | Not described |
| Syphilis | Antibiotics for syphilis diagnosed during pregnancy[159] | 0 | 0 |
| Tetanus prophylaxis | Vaccines for women to prevent neonatal tetanus [160] | 2 | 2 |
| Threat of premature delivery / Preterm labour | Antenatal corticosteroids for accelerating fetal lung maturation for women at risk of preterm birth [161] | 21 | 2 |
|  | Antenatal lower genital tract infection screening and treatment programs for preventing preterm delivery [162] | 1 | 0 |
|  | Bed rest in singleton pregnancies for preventing preterm birth [163] | 1 | 0 |
|  | Betamimetics for inhibiting preterm labour [164] | 17 | 0 |
|  | Calcium channel blockers for inhibiting preterm labour [165] | 12 | Not described |
|  | Cervical stitch (cerclage) for preventing pregnancy loss in women [166] | 6 | 1 |
|  | Combination of tocolytic agents for inhibiting preterm labour [167] | Protocol |  |
|  | Cyclo-oxygenase (COX) inhibitors for treating preterm labour [168] | 13 | Not described |
|  | Hydration for treatment of preterm labour [169] | 2 | Not described |
|  | Magnesium maintenance therapy for preventing preterm birth after threatened preterm labour [170] | 3 | 0 |
|  | Magnesium sulphate for preventing preterm birth in threatened preterm labour [171] | 29 | 3 |
|  | Maintenance therapy with calcium channel blockers for preventing preterm birth after threatened preterm labour [172] | 1 | Not described |
|  | Maintenance therapy with oxytocin antagonists for inhibiting preterm birth after threatened preterm labour [173] | Protocol |  |
|  | Nitric oxide donors for the treatment of preterm labour [174] | 5 | 1 |
|  | Oral betamimetics for maintenance therapy after threatened preterm labour [175] | 11 | 0 |
|  | Oxytocin receptor antagonists for inhibiting preterm labour [176] | 6 | 1 |
|  | Prenatal administration of progesterone for preventing preterm birth [177] | 6 | 1 |
|  | Probiotics for preventing preterm labour [178] | 2 | 0 |
|  | Progestational agents for treating threatened or established preterm labour [179] | Protocol |  |
|  | Prophylactic antibiotics for inhibiting preterm labour with intact membranes [180] | 11 | Not described |
|  | Prophylactic oral betamimetics for preventing preterm labour in singleton pregnancies [181] | 1 | 0 |
|  | Repeat digital cervical assessment in pregnancy for identifying women at risk of preterm labour [182] | Protocol |  |
|  | Terbutaline pump maintenance therapy after threatened preterm labor for preventing preterm birth [183] | 2 | 0 |
| Twin pregnancy | Hospitalisation and bed rest for multiple pregnancy [184] | 6 | 4 |
|  | Prophylactic oral betamimetics for reducing preterm birth in women with a twin pregnancy [185] | 5 | 2 |
| *Vaginal bleeding* |  |  |  |
| Abortion | Antibiotics for incomplete abortion [95] | 1 | 1 |
|  | Surgical procedures to evacuate incomplete miscarriage [186] | 2 | 1 |
|  | Chinese medicinal herbs for vaginal bleeding of medical abortion [187] | Protocol |  |
|  | Expectant care versus surgical treatment for miscarriage [92] | 5 | Not described |
| Ectopic pregnancy | Interventions for tubal ectopic pregnancy [88] | 35 | 5 |
| Molar pregnancy | nil |  |  |
| Placenta praevia | As above |  |  |
| Abruptio placentae | As above |  |  |
| Vaginal discharge | Antibiotics for treating bacterial vaginosis in pregnancy [188] | 15 | Not described |
| Vitamin A deficiency | Vitamin A supplementation during pregnancy [33] | 5 | 3 |
| Vaccination and pregnancy | Pneumococcal vaccination during pregnancy for preventing infant infection [189] | 3 | 2 |
|  | Vaccines for women to prevent neonatal tetanus [160] | 2 | 2 |

| **Guideline Name** | Emergency Field Handbook |  |  |
| --- | --- | --- | --- |
| **Date** | 2005 |  |  |
| **Developer** | UNICEF |  |  |
| **Guideline section** | **Potentially Relevant Cochrane Reviews** | **No. of trials in review** | **No. from developing setting** |
| Part 1: Initial Actions |  |  |  |
| 1.1 The first 72 hours | Nil |  |  |
| 1.2 The initial assessment | Nil |  |  |
| Part 2: Coordination |  |  |  |
| 2.1 Coordination | Nil |  |  |
| Part 3: Assessment and monitoring |  |  |  |
| 3.1 Assessment and monitoring | Nil |  |  |
| 3.2 Child rights monitoring, reporting and advocacy | Nil |  |  |
| Part 4: Special circumstances |  |  |  |
| 4.1 Negotiating with non-state entities | Nil |  |  |
| 4.2 Humanitarian-military relations | Nil |  |  |
| 4.3 Assisting displaced persons | Nil |  |  |
| 4.4 Natural disasters | Insecticide-treated bed nets and curtains for preventing malaria [190] | 22 | 22 |
|  | Indoor residual spraying for preventing malaria [191] | Protocol |  |
|  | Vaccines for measles, mumps and rubella in children [24] | 31 | Not described |
|  | Psychological therapies for the prevention and treatment of post-traumatic stress disorder in children and adolescents [192] | Protocol |  |
| Part 5: Programme Core Commitments |  |  |  |
| 5.1 Health and nutrition | Community-based supplementary feeding for promoting the growth of young children in developing countries [1] | 4 | 4 |
|  | Optimal duration of exclusive breastfeeding [13] | 22 | 11 |
|  | Vaccines for measles, mumps and rubella in children [24] | 31 | Not described |
|  | Vitamin A supplementation for breastfeeding mothers [38] | Protocol |  |
|  | Vitamin A for treating measles in children [193] | 8 | 6 |
|  | Vitamin A for preventing acute lower respiratory tract infections in children up to seven years of age [22] | 9 | 7 |
|  | Vitamin A for non-measles pneumonia in children [194] | 6 | 6 |
|  | Neonatal vitamin A supplementation for the prevention of mortality and morbidity in term neonates in developing countries [23] | Protocol |  |
|  | Vaccines for women to prevent neonatal tetanus [160] | 2 | 2 |
|  | Routine iron supplementation for preventing or treating iron-deficiency anaemia in children in malaria-endemic areas [4] | Protocol |  |
|  | Effects of routine oral iron supplementation with or without folic acid for women during pregnancy [40] | 40 | At least 15. Not well described |
|  | Folate supplementation in pregnancy [42] | Protocol |  |
|  | Zinc supplementation for improving pregnancy and infant outcome [31] | 17 | 13 |
|  | Zinc supplementation for the prevention of pneumonia in children aged 2 months to 59 months [29] | Protocol |  |
|  | Oral zinc for treating diarrhoea in children [195] | 18 | 17 |
|  | Deworming drugs for treating soil-transmitted intestinal worms in children: effects on growth and school performance [21] | 34 | 34 |
|  | Iodised salt for preventing iodine deficiency disorders [25] | 6 | 4 |
|  | Iodine supplementation for preventing iodine deficiency disorders in children [26] | 26 | 22 |
|  | Vitamin C supplementation in pregnancy [34] | 5 | 3 |
|  | Vitamin D supplementation in pregnancy [35] | 2 | 0 |
|  | Vitamin E supplementation in pregnancy [36] | 4 | 2 |
|  | Oral vitamin B12 versus intramuscular vitamin B12 for vitamin B12 deficiency [196] | 2 | 1 |
|  | Pyridoxine (vitamin B6) supplementation in pregnancy [197] | 5 | 0 |
|  | Treatments for iron-deficiency anaemia in pregnancy [37] | 17 | Not described |
|  | Support for breastfeeding mothers [12] | 34 | 9 |
|  | Interventions for promoting the initiation of breastfeeding [62] | 7 | 2 |
|  | Early skin-to-skin contact for mothers and their healthy newborn infants [70] | 30 | 7 |
|  | Interventions to prevent hypothermia at birth in preterm and/or low birthweight infants [71] | 6 | 1 |
|  | Separate care for new mother and infant versus rooming-in for increasing the duration of breastfeeding [64] | Protocol |  |
|  | Early additional food and fluids for healthy breastfed full-term infants [67] | Protocol |  |
|  | Lay health workers in primary and community health care [198] | 43 | 8 |
|  | Interventions to decrease the risk of mother-to-child transmission of HIV-1 through breast milk [11] | Protocol |  |
|  | Cup feeding versus other forms of supplemental enteral feeding for newborn infants unable to fully breastfeed [20] | 4 | 1 |
|  | Growth monitoring in children [30] | 2 | 2 |
|  | Postnatal parental education for improving family health [199] | Protocol |  |
|  | Traditional birth attendant training for improving health behaviours and pregnancy outcomes [14] | 4 | 4 |
|  | Specialist outreach clinics in primary care and rural hospital settings [200] | 9 | 1 |
|  | Patterns of routine antenatal care for low-risk pregnancy [201] | 10 | 3 |
|  | Physical methods for treating fever in children [202] | 7 | 1 |
|  | Antipyretic measures for treating fever in malaria [84] | 3 | 2 |
|  | Intramuscular arteether for treating severe malaria [47] | 2 | 2 |
|  | Sulfadoxine-pyrimethamine plus artesunate versus sulfadoxine-pyrimethamine plus amodiaquine for treating uncomplicated malaria [48] | 4 | 4 |
|  | Chlorproguanil-dapsone for treating uncomplicated malaria [49] | 6 | 6 |
|  | Artesunate plus mefloquine versus mefloquine for treating uncomplicated malaria [50] | 8 | 8 |
|  | Intra-rectal quinine for treating Plasmodium falciparum malaria [51] | 8 | 8 |
|  | Artesunate versus quinine for treating severe malaria [52] | 6 | 6 |
|  | High first dose quinine regimen for treating severe malaria [53] | 4 | 4 |
|  | Chloroquine or amodiaquine combined with sulfadoxine-pyrimethamine for treating uncomplicated malaria [54] | 4 | 4 |
|  | Artemether-lumefantrine (six-dose regimen) for treating uncomplicated falciparum malaria [55] | 9 | 9 |
|  | Artemether-lumefantrine (four-dose regimen) for treating uncomplicated falciparum malaria [56] | 7 | 7 |
|  | Atovaquone-proguanil for treating uncomplicated malaria [57] | 10 | 9 |
|  | Insecticide-treated bed nets and curtains for preventing malaria [190] | 5 | 5 |
|  | Oral versus intravenous rehydration for treating dehydration due to gastroenteritis in children [203] | 17 | 8 |
|  | Reduced osmolarity oral rehydration solution for treating dehydration caused by acute diarrhoea in children [204] | 13 | 12 |
|  | Antibiotic therapy for Shigella dysentery [205] | Protocol |  |
|  | Antibiotics for community acquired pneumonia in children [206] | 20 | At least 5. Not well described. |
|  | Short-course versus long-course antibiotic therapy for non-severe community-acquired pneumonia in children aged 2 months to 59 months [207] | 3 | 3 |
|  | Vitamin A for preventing acute lower respiratory tract infections in children up to seven years of age [22] | 9 | 7 |
|  | Vitamin C for preventing and treating pneumonia [208] | 5 | 0 |
|  | Oral antibiotics versus parenteral antibiotics for severe pneumonia in children [209] | 2 | 2 |
|  | Vitamin A for non-measles pneumonia in children [194] | 6 | 6 |
|  | Advising patients to increase fluid intake for treating acute respiratory infections [210] | 0 | 0 |
|  | Interventions for the interruption or reduction of the spread of respiratory viruses [211] | 51 | At least 5. Not well described. |
|  | Vaccines for women to prevent neonatal tetanus [160] | 2 | 2 |
|  | Vitamin C for preventing and treating tetanus [212] | 1 | 1 |
| 5.2 Water, sanitation and hygiene | Interventions to improve water quality for preventing diarrhoea [69] | 30 | 29 |
|  | Hand washing for preventing diarrhoea [44] | 14 | 6 |
|  | Interventions to improve excreta disposal for preventing diarrhoea [213] | Protocol |  |
|  | Face washing promotion for preventing active trachoma [45] | 2 | 2 |
|  | Environmental sanitary interventions for preventing active trachoma [46] | 4 | 4 |
| 5.3 Child protection | Psychological treatment of post-traumatic stress disorder (PTSD) [214] | 33 | 0 |
|  | Psychological debriefing for preventing post traumatic stress disorder (PTSD) [215] | 15 | 0 |
|  | Psychological therapies for the prevention and treatment of post-traumatic stress disorder in children and adolescents [192] | Protocol |  |
|  | Sports, games and play-based interventions for post-traumatic stress disorder (PTSD) [216] | Protocol |  |
|  | Multiple session early psychological intervention to prevent and treat post-traumatic stress disorder [217] | Protocol |  |
|  | Screening women for intimate partner violence in health care settings [218] | Protocol |  |
|  | Advocacy interventions to reduce or eliminate violence and promote the physical and psychosocial well-being of women who experience intimate partner abuse [219] | Protocol |  |
| 5.4 Education | Nil |  |  |
| 5.5 HIV/AIDS | Effectiveness and safety of HIV post-exposure prophylaxis after sexual, injecting-drug-use or other non-occupational exposure [220] | Protocol |  |
|  | Interventions for Educating Traditional Healers about STD and HIV Medicine [221] | Protocol |  |
|  | Interventions for improving the psychosocial wellbeing of children affected by HIV/AIDS [222] | Protocol |  |
|  | Home-based HIV voluntary counseling and testing in developing countries [223] | 2 | 2 |
|  | Rapid Tests vs. Traditional Tests for Screening of HIV infections [224] | Protocol |  |
|  | Behavioral interventions to modify sexual risk behaviors for preventing HIV infection in men who have sex with men [225] | 44 | 1 |
|  | Mass media interventions for promoting HIV testing [226] | 14 | 0 |
| Part 6: Operational Core Commitments |  |  |  |
| 6.1 Security | Nil |  |  |
| 6.2 Fund-raising and communication | Nil |  |  |
| 6.3 Human resources | Preventing occupational stress in healthcare workers [227] | 14 | Possibly 1. Not well described |
| 6.4 Information technology and telecommunications | Nil |  |  |
| 6.5 Supply and logistics | Nil |  |  |
| 6.6 Finance and administration | Nil |  |  |
| Part 7: Index |  |  |  |

| **Guideline Name** | Manual for the health care of children in humanitarian emergencies |  |  |
| --- | --- | --- | --- |
| **Date** | 2008 |  |  |
| **Developer** | WHO |  |  |
| **Guideline section** | **Potentially Relevant Cochrane Reviews** | **No. of trials in review** | **No. from developing setting** |
| Module 1 Triage and Emergency Management |  |  |  |
| Triage and emergency assessment | Nil |  |  |
| Management of emergency signs | Drug management for acute tonic-clonic convulsions including convulsive status epilepticus in children [228] | 4 | At least 1 |
| Module 2 Integrated management of childhood illness in emergencies |  |  |  |
| Diarrhoea and dehydration | Oral zinc for treating diarrhoea in children [195] | 18 | 17 |
|  | Reduced osmolarity oral rehydration solution for treating dehydration caused by acute diarrhoea in children [204] | 13 | 12 |
|  | Oral versus intravenous rehydration for treating dehydration due to gastroenteritis in children [203] | 17 | 8 |
|  | Antibiotic therapy for Shigella dysentery[205] | Protocol |  |
| Cough or difficulty breathing | Antibiotics for community acquired pneumonia in children [206] | 20 | At least 5. Not well described. |
|  | Short-course versus long-course antibiotic therapy for non-severe community-acquired pneumonia in children aged 2 months to 59 months [207] | 3 | 3 |
|  | Vitamin C for preventing and treating pneumonia [208] | 5 | 0 |
|  | Oral antibiotics versus parenteral antibiotics for severe pneumonia in children [209] | 2 | 2 |
|  | Vitamin A for non-measles pneumonia in children [194] | 6 | 6 |
|  | Advising patients to increase fluid intake for treating acute respiratory infections [210] | 0 | 0 |
|  | Holding chambers (spacers) versus nebulisers for beta-agonist treatment of acute asthma [229] | 33 | 15 |
|  | Bronchodilators for bronchiolitis [230] | 22 | At least 3. |
|  | Glucocorticoids for croup [231] | 31 | Not described |
| Fever | Intramuscular arteether for treating severe malaria [47] | 2 | 2 |
|  | Sulfadoxine-pyrimethamine plus artesunate versus sulfadoxine-pyrimethamine plus amodiaquine for treating uncomplicated malaria [48] | 4 | 4 |
|  | Chlorproguanil-dapsone for treating uncomplicated malaria [49] | 6 | 6 |
|  | Artesunate plus mefloquine versus mefloquine for treating uncomplicated malaria [50] | 8 | 8 |
|  | Intra-rectal quinine for treating Plasmodium falciparum malaria [51] | 8 | 8 |
|  | Artesunate versus quinine for treating severe malaria [52] | 6 | 6 |
|  | High first dose quinine regimen for treating severe malaria [53] | 4 | 4 |
|  | Chloroquine or amodiaquine combined with sulfadoxine-pyrimethamine for treating uncomplicated malaria [54] | 4 | 4 |
|  | Artemether-lumefantrine (six-dose regimen) for treating uncomplicated falciparum malaria [55] | 9 | 9 |
|  | Artemether-lumefantrine (four-dose regimen) for treating uncomplicated falciparum malaria [56] | 7 | 7 |
|  | Atovaquone-proguanil for treating uncomplicated malaria [57] | 10 | 9 |
|  | Antipyretic measures for treating fever in malaria [84] | 3 | 2 |
|  | Vitamin A for treating measles in children [193] | 8 | 6 |
|  | Fluid therapy for acute bacterial meningitis [232] | 3 | 2 |
|  | Antibiotics for acute otitis media in children [233] | 10 | 0 |
|  | Short course antibiotics for acute otitis media [234] | 32 | 5 |
| Malnutrition | Nil |  |  |
| Pallor/anaemia | Iron supplementation for iron deficiency anemia in children [3] | Protocol |  |
|  | Iron therapy for improving psychomotor development and cognitive function in children under the age of three with iron deficiency anaemia [2] | 7 | 4 |
|  | Blood transfusion for treating malarial anaemia[235] | 2 | 2 |
| Newborn and young infant up to 2 months | Prophylactic vitamin K for vitamin K deficiency bleeding in neonates[236] | 13 | At least 2. Not well described |
| HIV/AIDS | Nil |  |  |
| Injuries | Nil |  |  |
| Burns | Dressings for superficial and partial thickness burns [237] | Protocol |  |
| Poisoning | Oximes for acute organophosphate pesticide poisoning [238] | 2 | 2 |
|  | Alkalinisation for organophosphorus pesticide poisoning [239] | 1 | Not well described. |
|  | Interventions for paracetamol (acetaminophen) overdose [240] | 58 | At least 1. Not well described |
| Module 3 Prevention of child morbidity and mortality |  |  |  |
| Immunization and other public health measures | Interventions to improve water quality for preventing diarrhoea [69] | 30 | 29 |
|  | Interventions to improve excreta disposal for preventing diarrhoea [213] | Protocol |  |
|  | Hand washing for preventing diarrhoea [44] | 14 | 6 |
|  | Support for breastfeeding mothers [12] | 34 | 9 |
|  | Interventions for promoting the initiation of breastfeeding [62] | 7 | 2 |
|  | Vitamin A for preventing acute lower respiratory tract infections in children up to seven years of age [22] | 9 | 7 |
|  | Vaccines for measles, mumps and rubella in children [24] | 31 | Not described |
|  | Insecticide-treated bed nets and curtains for preventing malaria [190] | 22 | 22 |
|  | Indoor residual spraying for preventing malaria [191] | Protocol |  |
|  | Deworming drugs for treating soil-transmitted intestinal worms in children: effects on growth and school performance [21] | 34 | 34 |
|  | Optimal duration of exclusive breastfeeding [13] | 22 | 11 |
|  | Topical umbilical cord care at birth [241] | 21 | 1 |
|  | Kangaroo mother care to reduce morbidity and mortality in low birthweight infants [72] | 3 | 3 |
|  | Early skin-to-skin contact for mothers and their healthy newborn infants [70] | 30 | 7 |
|  | Interventions to prevent hypothermia at birth in preterm and/or low birthweight infants [71] | 6 | 1 |
| Prevention of HIV Infection in children | Community-based supplementary feeding for promoting the growth of young children in developing countries [1] | 4 | 4 |
|  | Vitamin A supplementation for reducing the risk of mother-to-child transmission of HIV infection [18] | 4 | 4 |
|  | Antiretrovirals for reducing the risk of mother-to-child transmission of HIV infection [16] | 14 | 14 |
|  | Interventions to decrease the risk of mother-to-child transmission of HIV-1 through breast milk [11] | Protocol |  |
|  | Vaginal disinfection for preventing mother-to-child transmission of HIV infection [19] | 2 | 2 |
| Mental health and psychosocial support | Psychological therapies for the prevention and treatment of post-traumatic stress disorder in children and adolescents [192] | Protocol |  |
|  | Psychological treatment of post-traumatic stress disorder (PTSD) [214] | 33 | 0 |
|  | Psychological debriefing for preventing post traumatic stress disorder (PTSD) [215] | 15 | 0 |
|  | Sports, games and play-based interventions for post-traumatic stress disorder (PTSD) [216] | Protocol |  |
|  | Multiple session early psychological intervention to prevent and treat post-traumatic stress disorder [217] | Protocol |  |
|  | Interventions for improving the psychosocial wellbeing of children affected by HIV/AIDS [222] | Protocol |  |
